# Genetic Association Between *NFKBIA* -881A>G Polymorphism and Cancer Susceptibility

**DOI:** 10.1097/MD.0000000000001024

**Published:** 2015-08-07

**Authors:** Peiliang Geng, Juanjuan Ou, Jianjun Li, Yunmei Liao, Ning Wang, Rina Sa, Lisha Xiang, Houjie Liang

**Affiliations:** From the Department of Oncology and Southwest Cancer Center, Southwest Hospital Third Military Medical University, Chongqing, China.

## Abstract

Several epidemiological studies have focused on the role of nuclear factor-kappa-B inhibitor-alpha (*NFKBIA*) -881 A>G polymorphism in cancer susceptibility. However, the published data have led to contentious results. This study was designed to examine the association between -881 A>G polymorphism and cancer risk.

Comprehensive search of PubMed, Web of science and Embase, identified a total of 5 case-control studies. To assess the association, comparison among all subjects plus subgroup analysis by ethnicity was performed and odds ratio (OR) along with 95% confidence interval (CI) was calculated with the fixed-effect model or the random-effects model dependent on the heterogeneity.

The pooling data consisting of 1965 cancer cases and 2717 cancer-free controls demonstrated no significant association with overall cancer risk. However, the subgroup of Asian populations showed statistical evidence for an increase in risk of cancer (GG vs. AA, OR, 2.14; 95% CI, 1.03–4.46; GG + GA vs. AA, OR, 1.22; 95% CI, 1.01–1.47; GG vs. GA + AA, OR, 2.09; 95% CI, 1.01–4.34).

This investigation on the association of -881 A>G polymorphism and cancer susceptibility reveals that -881 A>G polymorphism may act as a candidate for cancer development in Asian populations.

## INTRODUCTION

The transcription activator nuclear factor kappa-beta (NF-κB) controls the expression of various genes with involvement in cancer-related processes including immune and inflammatory response, cell adhesion, proliferation, differentiation, apoptosis, and angiogenesis.^1,2^ NF-κB comprises a multicomponent protein complex as a central mediator of many viral and cellular genes.^3^ It attaches to the cytoplasm of inactive cell lines by forming a complex with IκB inhibitor proteins (IκBα and IκBβ), which are responsible for nuclear localization of NF-κB in mammalian cells.^4,5^ It has been identified that phosphorylation of the IκB proteins causes rapid ubiquitination and subsequent proteolysis by 26S proteosome. Degradation of IκB proteins initiated by proteosome induces NF-κB dimers, resulting in accumulation of NF-κB in the nucleus and aberrant expression of specific target genes.^6^ Of the IκB family members (IκBα, IκBβ, IκBγ, IκBδ, IκBε, IκBζ, IκB-R, Bcl-3, p100, and p105) that can be found in cytoplasm and nucleus, all are constitutively expressed except that the typical form IκBα expressed induciblely.^7,8^ IκBα suppresses the activation of inflammation by binding to NF-κB in most normal cell types and plays a major role in circumventing oncogenesis due to its missing inhibitory function in a variety of cancer cells.^9–13^

Functioning as a candidate tumor suppressor gene involved in controlling the oncogenic potential of NF-kB, nuclear factor-kappa-B inhibitor-alpha (*NFKBIA*) precludes nuclear translocation, DNA binding, and phosphorylation by protein kinase alpha.^14^ The highly polymorphic *NFKBIA* gene at chromosome 14q13 spanning approximately 3.5 kb consists of 6 exons. Single nucleotide polymorphisms (SNPs) in the promoter region of *NFKBIA* are reported to be associated with the initiations of breast cancer, colorectal cancer, Hodgkin lymphoma, multiple myeloma, and melanoma.^4,15–18^ A commonly functional SNP located at GATA binding protein 2 is -881 A>G (rs3138053), which has been frequently implicated in hepatocellular carcinoma, colorectal cancer, ovarian cancer, and oral cancer.^19–23^ Since a single study using a sufficiently large sample size is currently unknown, which constitutes the main limitation of determining the association between -881 A>G polymorphism and cancer susceptibility. To clarify this issue, we performed a meta-analysis plus subgroup analysis from all eligible data associating the occurrence of cancer with -881 A>G polymorphism.

## METHODS

### Literature Search Strategy

Potentially relevant studies focusing on the association between -881 A>G polymorphism and cancer susceptibility published in English were searched using the databases of PubMed, Web of science and Embase. The key words *NFKBIA*, -881 A>G/rs3138053, polymorphism/polymorphisms/variant/SNP, and cancer were used in separation or in combination for the search strategy. The identification of additional original studies was carried out through screening the citations used in the papers that may have usable data for this meta-analysis. The study was approved by the ethics committee of southwest hospital.

### Inclusion and Exclusion Criteria

Eligibility of the studies for this meta-analysis was examined based on the following criteria: first, a case-control study looking at cancer susceptibility in relation to -881 A>G polymorphism; second, published as a full text detailing genotype data to calculate an odds ratio (OR) and corresponding 95% confidence interval (CI). Abstracts, unpublished reports, and articles written in non-English language were not considered. When the same case series were used in a subsequently published paper, we selected the study with more genotyped subjects.

### Data Extraction

Two authors independently gathered information from each study, including the first author's name, year of publication, ethnic origin of the participants (Asian or Caucasian), study design, cancer type, and genotype frequencies for AA, AG, and GG genotypes. For the disputable views on the extracted data, a senior reviewer was invited to resolve the differences.

### Statistical Analysis

Stata software (version 12.0, Stata Corp LP, College Station, TX) was performed to analyze all statistical data in the meta-analysis. Deviation from Hardy–Weinberg equilibrium (HWE) was checked for the genotype frequencies of controls in each study by using a *χ*^2^-test. An OR along with 95% CI was calculated through comparison among all subjects plus subgroup analysis by ethnicity to estimate cancer susceptibility associated with -881 A>G polymorphism. In addition to the Cochran Q-test, *I*^2^ statistic by Higgins and Thompson^24^ was quantified to examine heterogeneity between studies, *P* < 0.10 or *I*^2^ > 50% being considered statistically significant. The fixed-effects model using the Mantel–Haenszel method^25^ was performed for OR estimate if *P* > 0.10 or *I*^2^ < 50%. Conversely, the random-effects model using the DerSimonian Laird method was used.^26^ Sensitivity analysis was performed to detect the stability of the meta-analysis results by removing the studies, one at a time. Funnel plots and Egger test^27^ were used to determine the publication bias. Symmetrical funnel plots and *P* values of the Egger test above 0.10 revealed little significant publication bias across studies.

## RESULTS

### Characteristics of Included Studies

**FIGURE 1:**
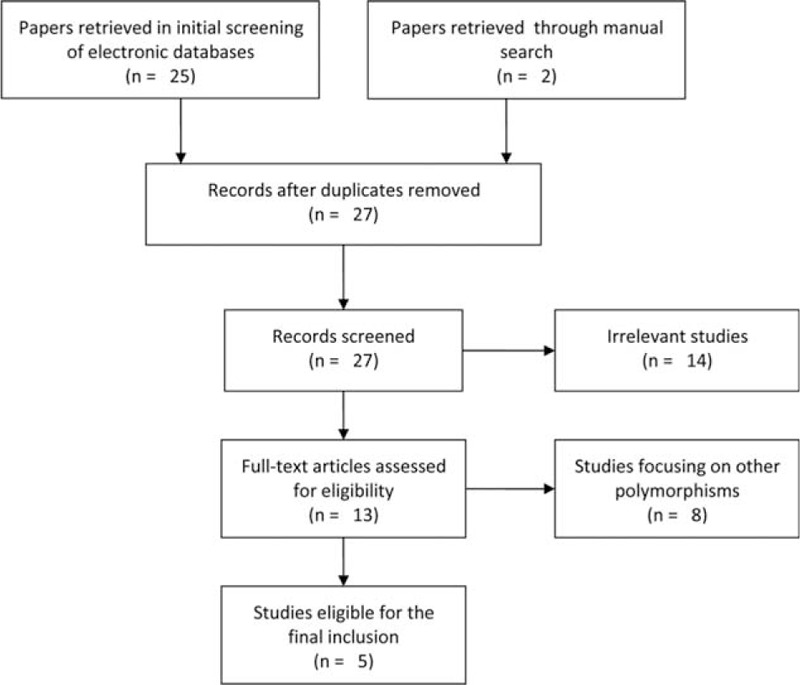
Flow diagram of the study selection process.

A total of 27 relevant papers were retrieved from initial screening. Among these, 13 full texts remained for eligibility evaluation, of which 8 publications were finally excluded for focusing on cancer risk and other polymorphisms of *NFKBIA* gene.^4,15–17,28–31^ Therefore, our final data pooling consisted of 5 case-control studies^19–23^ involving 1965 cancer cases and 2717 cancer-free controls (Figure 1).

Overall, there were 2 hepatocellular carcinoma studies, 1 prostate cancer study, 1 ovarian cancer study, and 1 oral cancer study. Of the 5 case-control studies, 4 were for the subjects of Asian ancestry and 1 for Caucasian ancestry. Moreover, all publications were in line with HWE except the study by He et al (Table 1).

**TABLE 1:**
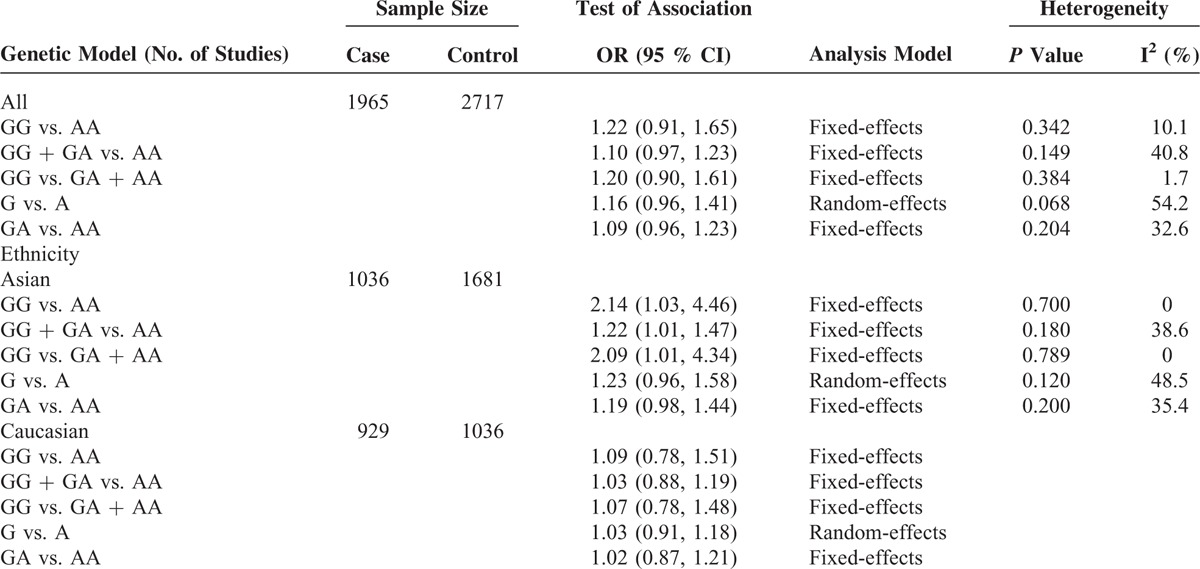
Characteristics of Studies Included in the Meta-Analysis

### Quantitative Synthesis

Association of -881 A>G polymorphism and cancer susceptibility was assessed in pooling data of 5 case-control studies including 1965 cancer cases and 2717 cancer-free controls. Overall, none of the comparison models showed significant association with cancer susceptibility. The pooled ORs were not significantly altered when the analysis was restricted to the studies in accordance with HWE.

**FIGURE 2:**
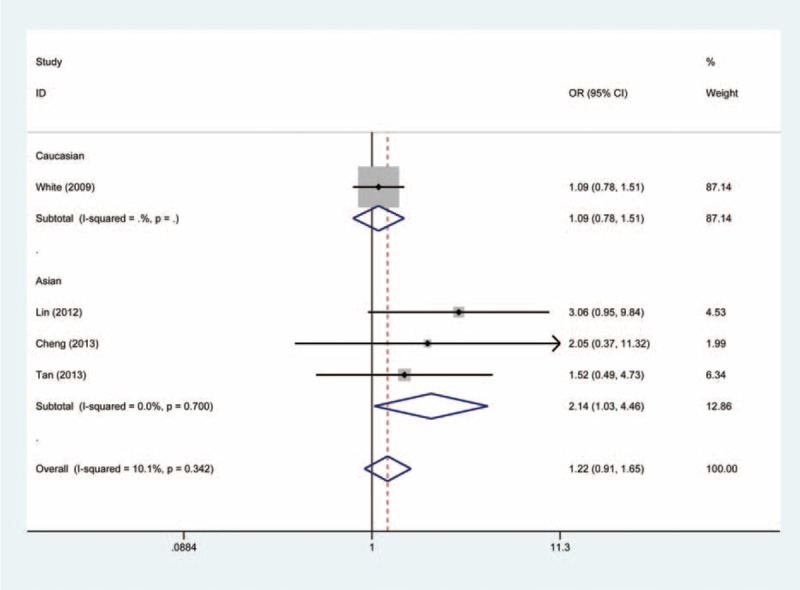
Forest plot of the association between *NFKBIA* -881 A>G polymorphism and cancer susceptibility under GG vs. AA genetic model.

**FIGURE 3:**
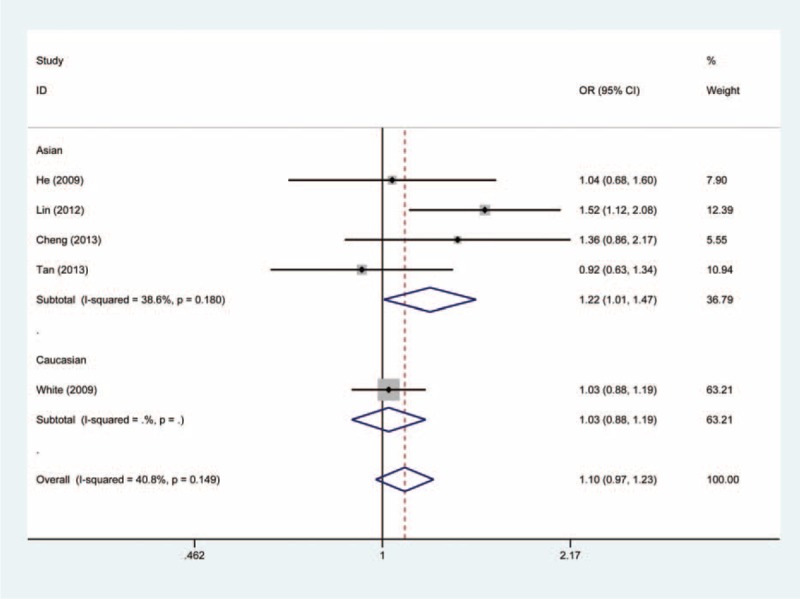
Forest plot of the association between *NFKBIA* -881 A>G polymorphism and cancer susceptibility under GG + GA vs. AA genetic model.

**FIGURE 4:**
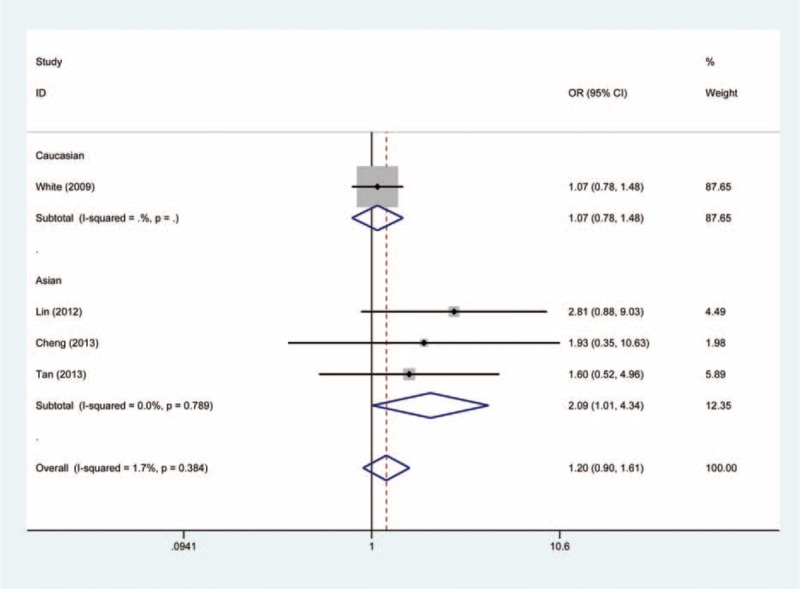
Forest plot of the association between *NFKBIA* -881 A>G polymorphism and cancer susceptibility under GG vs. GA + AA genetic model.

In the stratified analysis, however, the subgroup of Asian populations demonstrated an increased risk of cancer. The OR of the GG genotype was 2.14 (GG vs. AA, OR, 2.14; 95% CI, 1.03–4.46) compared with the AA genotype (Figure 2). The carriers of GG + GA genotypes had 1.22-fold risk of developing cancer relative to the AA genotype carriers (GG + GA vs. AA, OR, 1.22; 95% CI, 1.01–1.47) (Figure 3). In addition, as compared to the GA + AA genotypes, the OR of the GG genotype was 2.09 (GG vs. GA + AA, OR, 2.09; 95% CI, 1.01–4.34) (Figure 4). However, we observed no increased or decreased risk in Caucasian populations (Table 2).

**TABLE 2:**
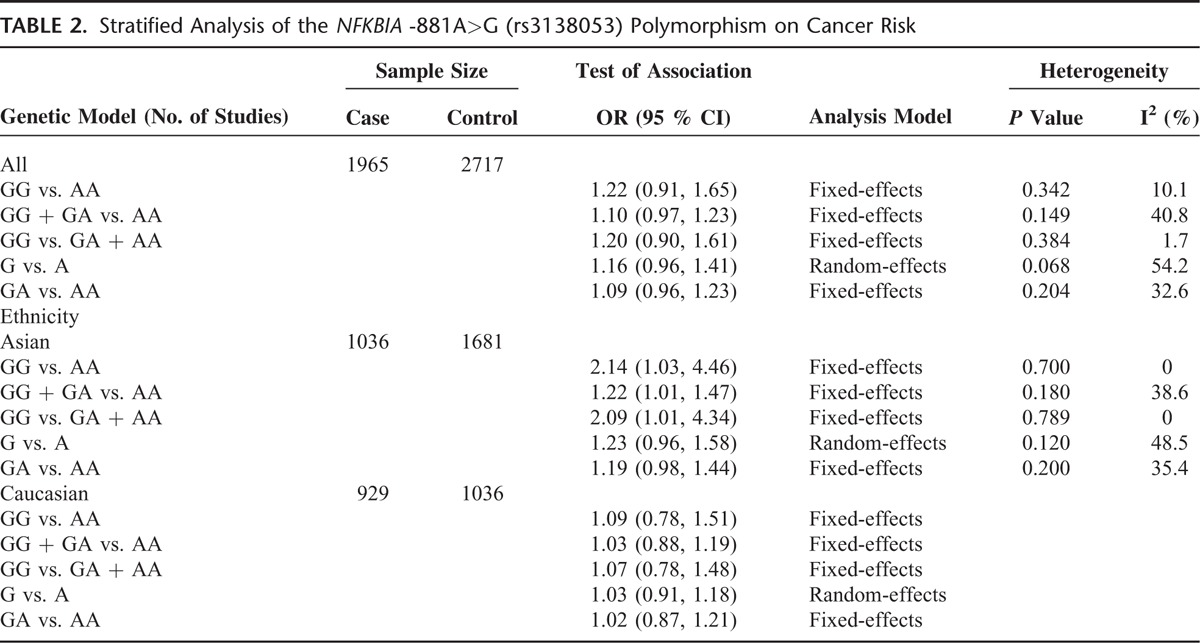
Stratified Analysis of the *NFKBIA* -881A>G (rs3138053) Polymorphism on Cancer Risk

In the present study, heterogeneity across studies was evaluated by Q test and the *I*^2^ index, where significant between-study heterogeneity was indicated in the allele model (G vs. A, *P*, 0.068; *I*^2^, 54.2%) (Table 2). Subgroup analysis by ethnicity and subsequent sensitivity analysis restricted the main source to the study by White and co-workers.^20^ Intriguingly, when removing this study from the total meta-analysis, heterogeneity was lost (*P*, 0.120; *I*^2^, 48.5%) and additionally this genetic model was found to be significantly associated with cancer risk (before removal, OR, 1.16; 95% CI, 0.96– 1.41; after removal, OR, 1.25; 95% CI, 1.05–1.49).

### Publication Bias

**FIGURE 5:**
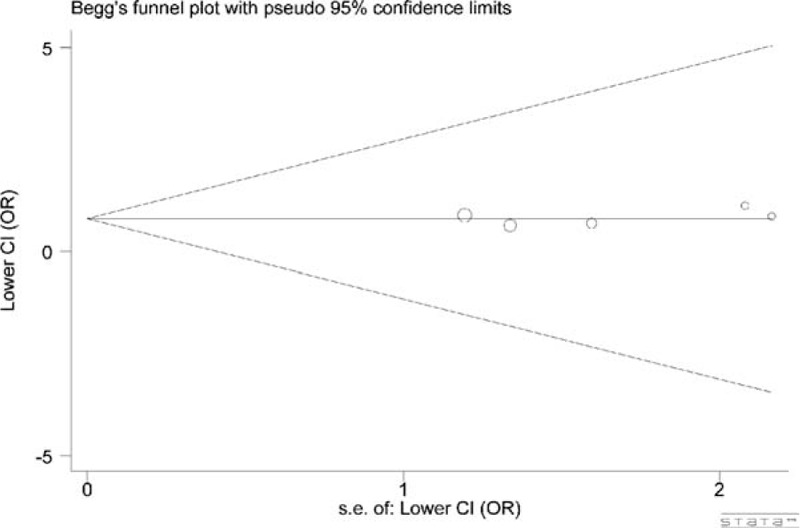
Funnel plots of *NFKBIA* -881 A>G polymorphism and cancer risk. *P*, Begg test = 0.860; *P*, Eager test = 0.521; model, GG + GA vs. AA.

We performed Begg test and Egger test to diagnose the publication bias of all included studies. The symmetrical funnel plot (*P*, 0.860) and Egger test (*P*, 0.521) revealed no statistical evidence for significant publication bias in this meta-analysis (for GG + GA vs. AA) (Figure 5).

## DISCUSSION

To the best of our knowledge, our study is the available meta-analysis comprehensively investigating for the first time cancer susceptibility associated with -881 A>G polymorphism in the promotor region of the *NFKBIA* gene. It is believed that a study of predisposition genetic polymorphisms with a large sample size is capable of supplying deeper insight into the association between candidate genes and diseases. With 5 case-control studies containing 1965 cancer cases and 2717 healthy controls, we found that -881 A>G polymorphism was not associated with overall cancer risk. Interestingly, analysis in subgroups of Asian and Caucasian subjects revealed significantly elevated risk of cancer in Asian populations, but not in Caucasian populations.

Genetic variations in genes modulate the initiation and progression of cancer. IκBα belonging to IκB family is encoded by the *NFKBIA* gene and confers inhibitory function on NF-κB, a set of pleiotropic transcription factors playing central roles in inflammation and innate immune responses connected with carcinogenesis.^32,33^ Proinflammatory cytokines-dependent activation stimulates NF-κB complexes mediating the expression of genes involved in apoptosis, angiogenesis, and cell growth.^34,35^ Due to the central role played in immunological processes, NF-κB has attracted widespread attention in many fields and its abnormal activation has been shown to be linked with several diseases (autoimmune arthritis, asthma, septic shock, lung fibrosis, atherosclerosis, and AIDS).^36,37^

In the past few years, -881 A>G polymorphism of the *NFKBIA* gene has been a research focus in many cancer communities. Nevertheless, the results produced great disparities; such differences existed even in the same cancer. In more detail, both He et al^19^ and Cheng et al^22^ concerning hepatocellular carcinoma demonstrated discoveries very different with each other. It held true for other cancers. Oral cancer was revealed to be modulated by -881 A>G polymorphism individually and jointly with *NFKB1* -94 ATGG2/ATGG2 polymorphism.^21^ However, colorectal cancer was observed to be decreased in women rather than in men.^23^ There are 2 explanations for this inconsistency. First, the limited number of subjects used in each individual investigation lacks statistical power to identify the relationship of -881 A>G polymorphism and cancer susceptibility. Second, they are not conducted in the same populations. Different genetic backgrounds and lifestyles as a result of diverse ethnic origins could lead to an incorrect observation which misleads further replicated studies. Given the importance of -881 A>G polymorphism in cancer-related pathways and the existing contentious association with cancer, we examined the correlation by performing a meta-analysis, where we found significantly increased cancer risk in Asian populations.

The finding of our meta-analysis should be interpreted with caution. We observed significant heterogeneity in the allele model and found the major source of the heterogeneity masked the true association between this comparison with risk of cancer. Although no obvious inter-study heterogeneity was indicated in the Q-test and *I*^2^ statistic under other genetic models, we cannot exclude the possibility that our findings have been affected by potential heterogeneity across studies. Furthermore, caner risk was not shown to be related to -881 A>G polymorphism in Caucasian populations. In the current study, only 1 publication based on Caucasians was included, which may have underestimated the association. Then, the susceptibility of -881 A>G polymorphism to certain cancers has been suggested to be different in women and men. Therefore, other confounding factors, such as sex, should be taken into account in future studies if sufficient data are provided.

To sum up, this meta-analysis of the association between -881 A>G polymorphism and cancer susceptibility suggested that -881 A>G polymorphism did not modulate overall cancer risk. However, we found statistical evidence for an increased risk of cancer in Asian populations. Further large sample-sized studies are necessary to provide new insights into the mechanism of cancer development related to -881 A>G polymorphism.
